# The role of interoceptive inference in theory of mind

**DOI:** 10.1016/j.bandc.2015.08.002

**Published:** 2017-03

**Authors:** Sasha Ondobaka, James Kilner, Karl Friston

**Affiliations:** Wellcome Trust Centre for Neuroimaging, University College London, United Kingdom; Sobell Department for Motor Neuroscience and Movement Disorders, University College London, United Kingdom

**Keywords:** Free energy, Predictive processing, Emotion, Intention, Expectation

## Abstract

Inferring the intentions and beliefs of another is an ability that is fundamental for social and affiliative interactions. A substantial amount of empirical evidence suggests that making sense of another’s intentional and belief states (i.e. theory of mind) relies on exteroceptive (e.g. visual and auditory) and proprioceptive (i.e. motor) signals. Yet, despite its pivotal role in the guidance of behaviour, the role of the observer’s interoceptive (visceral) processing in understanding another’s internal states remains unexplored. Predicting and keeping track of interoceptive bodily states – which inform intentions and beliefs that guide behaviour – is one of the fundamental purposes of the human brain. In this paper, we will focus on the role of interoceptive predictions, prescribed by the free energy principle, in making sense of internal states that cause another’s behaviour. We will discuss how multimodal expectations induced at deep (high) hierarchical levels – that necessarily entail interoceptive predictions – contribute to inference about others that is at the heart of theory of mind.

## Highlights

• Own mental states depend on inferred interoceptive states.
• Understanding another’s mental states (ToM) also entails interoceptive predictions.
• Multimodal expectations at deep hierarchical levels contribute to ToM.

## 1 Understanding others’ minds

### 1.1 Mechanisms for inferring others’ minds

Understanding or inferring of another’s intentions, feelings and beliefs is a hallmark of human social cognition often referred to as mentalising or having a Theory of Mind (ToM; Frith and Frith, 1999, Saxe and Kanwisher, 2003). ToM has been described as a cognitive ability to infer the mental states (intentions and beliefs) of others, through processing of their physical appearance and overt behaviour (e.g. clothes, bodily and facial expressions). Typically, neuronal computations underlying ToM have been associated with multimodal brain regions like the superior temporal sulcus (STS), temporoparietal junction (TPJ) and medial frontal cortex (MFC; Frith and Frith, 2006, Saxe, 2006). However, the nature of processing in higher multimodal regions that accumulates information from different streams remains poorly understood.

### 1.2 Predictive mechanisms in theory of mind

Recent views propose that predictive mechanisms could play a role in ToM (Hohwy, 2013, Kilner and Frith, 2008, Koster-Hale and Saxe, 2013). In brief, hypotheses about the intentions of others are tested against their observed behaviour by generating top-down predictions of that behaviour – and updating competing hypotheses on the basis of ensuing prediction error. Crucially, the repertoire of hypotheses that can be entertained is borrowed from the constructs (hypotheses) that cause one’s own behaviour. This provides a nice explanation for the role of the “mirroring mechanism” (Rizzolatti & Sinigaglia, 2010) in action observation and theory of mind – and the integration of multimodal data inherent in descending multimodal predictions (Kilner and Frith, 2008, Ondobaka and Bekkering, 2013, Ondobaka et al., 2014).

**Fig. 1:**
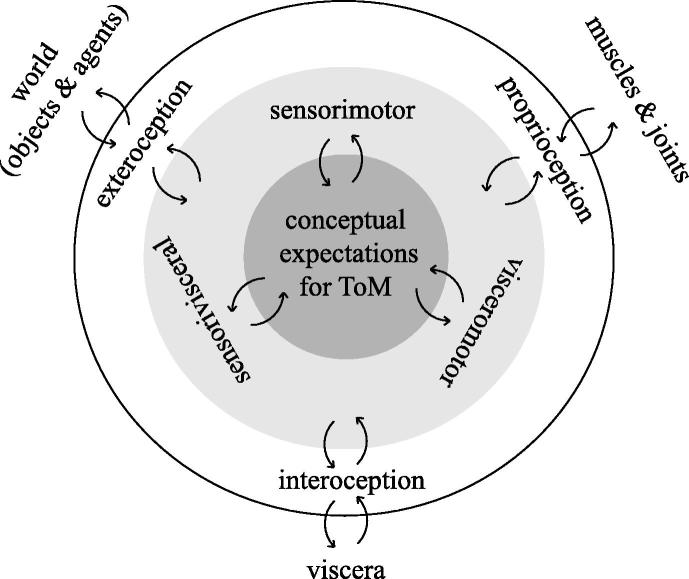
Schematic representation of a hierarchical predictive neural model for ToM that includes interoception, exteroception and proprioception. White-to-dark grey colour scale represents the neural hierarchy, in which conceptual expectations (dark grey) that include interoception sit high (deep) in the hierarchy. Arrows indicate hierarchical message passing in forward and backward directions carrying prediction error and expectation/prediction, respectively.

However, this perspective only addresses the predicted consequences of movement and says little about the predicted consequences of internal bodily states that contextualise behaviour. In other words, it is unclear how processing of internal visceral/autonomic information (interoception) could contribute to the understanding of others’ intentions. There are two ways of thinking about the role of interoception in ToM. The first relates to how processing of exteroceptive information about another’s interoceptive state helps us to infer states of mind that cause their behaviour. The second rests on how knowing the interoceptive causes of our own behaviour helps us predict and infer another’s. In this paper, we consider interoceptive inference as a special case of active inference, under the free energy principle (Friston, 2010) – and emphasise its potentially fundamental role in grounding the process of inferring another’s state of mind from their perceived (motor and autonomic) behaviour (Fig. 1).

## 2 The free energy and interoceptive inference

### 2.1 The free energy principle

The free energy principle requires the brain to generate continuous predictions in order to achieve its goal of minimizing free energy – an information theoretical quantity that reflects surprise or prediction error (Friston, 2009). Prediction errors are the difference between sensations and predictions of those sensations based upon an internal or generative model. The basic idea is that the brain constitutes a hierarchical generative model, through which the wealth of incoming information from the viscera, musculoskeletal system and the outside world is interpreted. This interpretation corresponds to inferring the causes of sensations in terms of representations or expectations that would generate the same sensory information, under the hierarchical model. Inferring the causes of visceral/autonomic, motor and sensory (e.g. visual, auditory) information corresponds to interoception, proprioception and exteroception, respectively. It is assumed that all the sensory streams in the brain are organised in a hierarchical fashion, with areas that sit higher (deeper) in the hierarchy representing more abstract information and generating expectations of lower levels (Ondobaka & Bekkering, 2013). At the apex of unimodal sensory hierarchies, multimodal brain regions encode conceptual expectations that necessarily accumulate unimodal information – or prediction errors (Fig. 1; Barrett, 2014, Ondobaka et al., 2014).

### 2.2 Two modes of free energy minimisation

Minimisation of free energy (prediction error) can occur in two distinct modes, we can either change our predictions to match sensations or we sample sensations to match predictions. Predictive coding typically refers to changing our predictions to match sensations (Friston, 2005, Rao and Ballard, 1999). Controlling motor and autonomic (visceral) system to experience sensations that match our predictions is known as active inference (Friston et al., 2011, Joffily and Coricelli, 2013). These complementary modes of minimising (exteroceptive and proprioceptive/interoceptive) prediction errors correspond to what we generally view as perception or inference and action or motor/autonomic control respectively. It is crucial to note here that two modes of free energy minimisation exist in both proprioceptive and interoceptive domains.

Perception minimises free energy by concurrent dynamical updating of expectations about the causes of external (exteroceptive) and internal (interoceptive and proprioceptive) sensory inputs. For example, perception of a surprising object is associated with an attempt to suppress visual prediction error (Rao & Ballard, 1999). Action, on the other hand, minimises prediction error by directly altering sensory inputs through movement and visceral control that fulfil proprioceptive and interoceptive predictions. For example, movement of the arm is driven by classical motor reflects arcs in the spinal-cord to suppress proprioceptive prediction error – such that descending proprioceptive predictions become motor commands that are reflexively executed by striated muscles (Adams, Shipp, & Friston, 2013). Similarly, the intensity and frequency of on-going contractions of the heart muscle can be modulated to suppress the interoceptive prediction error signalling surprising interoceptive states related to e.g. blood pressure (e.g. Kumagai et al., 2012). This reflexive suppression of interoceptive prediction error corresponds to autonomic reflexes mediated by smooth muscles.

### 2.3 Free energy and experience of intention and emotion

Proprioceptive and interoceptive prediction errors (free energy) used in motor and autonomic control might relate to our experience of intention and emotion (Seth, 2013, Shipp et al., 2013). Recent accounts of interoceptive inference have proposed that emotion could be understood from the perspective of hierarchical interoceptive inference (Joffily and Coricelli, 2013, Seth, 2013). For example, Seth (2013) views emotional content as the product of active inference about the likely internal and external causes of visceral changes. Joffily and Coricelli (2013) associated the rate of change of interoceptive prediction error with emotional valance, such that a shift from less expected/valued state (i.e. high free energy) to a more expected/valued state (i.e. low free energy) leads to positive valence. Conversely, negative valance corresponds to a shift from a low free energy to a high free energy state. Although the primary drive for motor and autonomic (visceral) control are descending proprioceptive and interoceptive predictions, these predictions are contextualised by deep hierarchical models that are necessarily accountable to conceptual representations that also generate exteroceptive predictions (Barrett, 2014, Barrett and Simmons, 2015). This enables the integration of exteroceptive information to contextualise (adaptive) motor and visceral responses. For example, when my interoceptive prediction errors signal hunger, I intend (expect) to move my arm to open the fridge because this is what I normally do when I feel (infer myself to be) hungry and find myself in the kitchen. We assume that these deep multimodal levels of representation that guide own behaviour play an important role in inference of others’ intentions and emotion.

## 3 Inferring another’s intentions and emotions

### 3.1 Interoceptive inference mechanism

Interoception refers to one of the fundamental purposes of the human brain – to predict and maintain internal bodily states within physiological bounds and relatively constant over time (Craig, 2009, Critchley and Harrison, 2013). Interoception or interoceptive inference can be viewed as a generalisation of active inference (Clark, 2013, Friston, 2010) to the processing of interoceptive signals carrying information about visceral states (e.g. heart rate, blood pressure, temperature). For example, recent computational work by Joffily and Coricelli (2013) suggests that rate of change of interoceptive free energy corresponds to experience of emotional valance and dynamical adaptation of behaviour. The interoceptive (visceral) processing hierarchy comprises of the brainstem nuclei (e.g., nucleus of the solitary tract, parabrachial nucleus, and the central grey) carrying afferent inputs to the thalamus, hypothalamus, the amygdala and the neocortex (Craig, 2009, Kumagai et al., 2012). Cortical regions associated with interoceptive processing include the cingulate gyrus, insula, somatosensory cortex, medial and orbital frontal cortex. We assume that interoceptive information encoded by mechanical, chemical and thermal receptors is processed in the light of top-down predictions generated by neuronally encoded expectations at each level of the interoceptive hierarchy (Friston, 2010). Mechanistically, the recurrent message passing between different levels in the interoceptive hierarchy will aim to minimise prediction errors at each level. This is exactly the same predictive coding scheme proposed for predictive processing of exteroceptive (Friston, 2005, Rao and Ballard, 1999) and proprioceptive (Adams et al., 2013, Shipp et al., 2013) information and applied to mechanisms that underlie understanding of intentions in others (Kilner et al., 2007, Ondobaka and Bekkering, 2013).

### 3.2 Interoceptive inference in ToM

In this paper, we suggest that delineating the interplay between interoceptive, proprioceptive and exteroceptive sensory processing is pivotal to understanding mechanisms that underlie ToM. We hypothesise that interoception, formulated under active inference, plays a fundamental role in ToM. From the active inference perspective, knowing the contents of another’s mind can be demystified and simply recast as an optimal explanation for perceived (motor and visceral) behaviour in others – that would have been produced by ourselves, have we had been in the same intentional and emotional state.

Note that deep generative models permit inferences at multiple levels. For example, inferring the interoceptive (visceral) states of another necessarily constrains the hypothesis space of plausible explanations for their current behaviour (e.g. she wants to go back indoors because she’s cold). Observer’s predictions of their own interoceptive states that cause a feeling of being cold play a principal role in understanding this feeling when seeing someone shiver. This is implied because interoceptive predictions are generated in the observer’s deep hierarchical model that concurrently generates exteroceptive predictions of seeing someone shiver. In this instance, the observation of shivering may induce an interoceptive or emotional contagion and empathy – implying that an observer can also be sympathetic to another’s desires and intentions (e.g. to go back into the warmth). Clearly, this form of deep vicarious active inference requires the attenuation of (proprioceptive and interoceptive) prediction errors that would otherwise cause echopraxia or overt emotional contagion (Hess and Fischer, 2014, Heyes, 2011).

### 3.3 Contrasting interoception to exteroception and proprioception

To understand the fundamental role of interoception in ToM, we might consider its unique contribution to (social) cognition by contrasting it to proprioceptive and exteroceptive inference. First, compared to proprioception and exteroception, interoceptive sensations have a low degree of spatiotemporal acuity and do not typically reach conscious awareness. For example, it is difficult to localise a stomach ache in space and time. Similarly, there is little conscious access to the peristaltic contractions of the transverse colon or status of renal function. These low levels of acuity or resolution are a direct consequence of the generative models we have inherited to predict the continuously changing interoceptive signals reported by a large variety of receptors. Several interesting predictions arise from formal or structural differences in the generative models for interoception, relative to visual or tactile cues. First, it suggests that we would be unable to integrate localised and transient cues of an interoceptive nature into beliefs about the emotional intentions of another. For example, a brief (one second) blush localised to one side of the face may be perceived as a brief change in ambient illumination, as opposed to a change in emotional state. Second, due to a high degree of exteroceptive–proprioceptive (i.e. visuomotor) correspondence, the effects of observed agent’s actions can be directly mapped onto the observer’s proprioceptive system (Rizzolatti & Sinigaglia, 2010). For example, both observer and the observed agent, can directly access exteroceptive information about the precise motor trajectories aimed at objects in the shared environment (Bach, Peelen, & Tipper, 2010). This exteroceptive information can then be combined with observer’s proprioceptive models to infer movement intentions (Ondobaka et al., 2014).

In contrast, interoceptive–exteroceptive correspondence is rather poor, making the direct mapping of others’ interoceptive states to our own interoception more challenging. In other words, there are far fewer interoceptive cues that can be observed (through exteroception) and consequently a much greater reliance upon counterfactual inference afforded by priors in generative models of our own interoceptive states. Similar to already proposed proprioceptive counterfactuals (Friston et al., 2012, Seth, 2015), interoceptive counterfactuals or simulations can be viewed as expectations or hypotheses about future sensory states that are conditioned upon certain interoceptive states. Interoceptive counterfactuals rest upon deep hierarchical models that extend into the future and entail both exteroceptive and proprioceptive expectations.

#### 3.3.1 From interoceptive inference to ToM

Whereas majority of the investigations on intentional and emotional aspects of ToM focus on exteroceptive and proprioceptive processing, the role of interoceptive processing has been somewhat neglected. Interoceptive states are informing and contextualizing behaviour by biasing perception and action towards fulfilling organism’s physiological needs. At a low level of the hierarchy this corresponds to homeostasis, while higher levels provide contextual guidance for allostasis (Barrett, 2013, Gu and FitzGerald, 2014). In a similar vein, Seth (2013) has proposed a fundamental role for interoception in body ownership, emotion and selfhood. Crucially, in exactly the same way that the proprioceptive mirroring mechanism may reflect a re-purposing of generative models for inferring and causing one’s own motor behaviour to infer another’s, it has also been proposed that interoceptive inference can also be deployed to infer the interoceptive states of others (particularly in theoretical treatments of autism: e.g., Quattrocki & Friston, 2014). In other words, in the same way that we simulate how we would move given a particular intention – to explain how someone else is moving, we may simulate our bodily states corresponding to a particular feeling to understand how someone else is feeling.

While interoceptive inference has also been associated with processing of other individuals’ emotional and feeling states (Singer et al., 2004, Wicker et al., 2003), the predictive mechanisms that integrate interoceptive information with visual and proprioceptive processing streams to model or read others’ minds remain unclear. Recent work provides behavioural evidence for the role of interoception in social inference by showing that ability to recognise others’ emotion from facial expression is correlated with perceivers’ ability to report their own interoceptive states (Cook, Brewer, Shah, & Bird, 2013). This mismatch between precise information about another’s motor behaviour, relative to their autonomic responses, speaks to the important role of (Bayesian) inference in reconciling multimodal cues to make inferences. It suggests that we will weight evidence about motor behaviour over evidence for changes in autonomic status. This differential weighting suggests a useful perspective on theory of mind, from the perspective of predictive multisensory integration. For example, what would be the effect of incongruent cues from another’s motor and autonomic responses (i.e., a gentle affiliative touch accompanied by piloerection and pupillary dilation)? Would these lead to a sense of uncertainty about the actor’s real intent or would the motor behaviour predominate? In principle, these questions can be answered empirically using behavioural or neuroimaging techniques.

#### 3.3.2 Differences between inferring mental and physical states

We have framed inference about the emotional states of others as a form of interoceptive mirroring mechanism (Singer et al., 2004, Wicker et al., 2003); however, there are clear differences between proprioceptive and interoceptive inference. A key difference is that the motor behaviour of another is readily accessible through visual and other exteroceptive cues. This is not necessarily the case for interoception; in the sense that many bodily states are hidden from (sensory) view. One might therefore ask is there are fundamental differences between inferring the behaviour of any inanimate object and inferring the internal state of another person. For motor behaviour, this is an easy question to answer because one can use one’s own intentional expectations and sensorimotor predictions to furnish a simple explanation for the behaviour of another (provided one applies a suitable perspective taking transformation to visual cues). Same proprioceptive generative models are used in inference of external physical events (Schubotz, 2007). In other words, instead of having to learn a generative model of another’s goal-directed behaviour, it is only necessary to repurpose one’s own generative models that are acquired during early neural development.

However, for interoceptive cues (in the exteroceptive modality), this perspective taking is more difficult and sometimes perhaps impossible. For example, we cannot see our pupils dilate. This suggests that the emotional and intentional theory of mind has to be learned through interpersonal interactions, probably at an early stage of development, in which attachments are made. However, if one can learn to explain or associate cues about the interoceptive state of another in terms of one’s own interoceptive state, all the deep hierarchical contingencies that underlie allostatic behaviour become available to predict what the other person may do next – and why they are doing it. It is in this sense that there may be something special about how we are particularly adept at inferring the drives and affiliative imperatives that contextualise interoception.

#### 3.3.3 Conceptual (multimodal) inference in ToM

During social inference, ToM or mentalising regions combine interoceptive information with proprioceptive and exteroceptive signals (Brass et al., 2009, Hayden et al., 2008, Pearson et al., 2011). Whereas the effects of one’s interoceptive states play a crucial role in behaviour (Critchley & Harrison, 2013), there is no direct one-to-one mapping of interoception onto proprioceptive and exteroceptive states. Interoceptive states are rather mapped, with exteroceptive and proprioceptive states onto multimodal constructs in a hierarchical fashion (Fig. 1). This means, each modality contextualises the others, through ascending prediction errors and resulting updates at deep or higher conceptual levels of the hierarchical model. For example, to maintain an expected heart rate, many (probabilistic) mappings to exteroceptive states (objects) and proprioceptive states (movements) could exist. Similarly, no one-to-one mapping exists between the agent’s and the observer’s interoceptive states. This pleiotropic (producing more than one effect) mapping precludes exact inference about another’s interoceptive states; e.g., their heart rate. Empirically speaking this form of vicarious (interoceptive) inference suggests that neuronal responses to changes in interoceptive cues should depend upon exteroceptive cues at and only at higher/conceptual levels of the hierarchy (e.g., cingulate, insular or prefrontal cortex). On the other hand, the fundamental advantage, offered by the probabilistic and pleiotropic mappings, is the potential to engage in simulation or counterfactual inference (Seth, 2015). Pleiotropic and probabilistic mappings between interoceptive and exteroceptive/proprioceptive states comfortably accommodate counterfactual conceptual inference – a central component of ToM. In principle, this sort of prediction can be tested using factorial neuroimaging experiments in which exteroceptive and interoceptive cues are manipulated orthogonally to elicit interactions in multimodal areas that generate top-down interoceptive predictions.

## 4 Conclusion

In summary, the inference about or understanding of models that cause another’s behaviour in mentalising or ToM may be mediated by joint minimisation of hierarchical prediction errors (free energy) elicited by unexpected sensations. Multimodal expectations induced at deep (high) hierarchical levels – that necessarily entail interoceptive predictions – should play a fundamental role in inferring causes of sensory impressions produced by the behaviour of other people. Most current social cognition models suggest that exteroceptive (e.g. visual and auditory) and proprioceptive (i.e. motor) processing underlie inference about intentional and emotional states. Embedding interoception in a multimodal active inference framework may offer a more complete and plausible model of ToM. This extension may also provide a principled account of ToM that appeals to a sense of agency and selfhood – and that is equipped with the interoceptive aspects of affect and feeling states.
